# Decline in Antigenicity of Tumor Markers by Storage Time Using Pathology Sections Cut From Tissue Microarrays

**DOI:** 10.1097/PAI.0000000000000172

**Published:** 2016-03-10

**Authors:** Fiona M. Blows, Hamid R. Ali, Sarah-J. Dawson, John Le Quesne, Elena Provenzano, Carlos Caldas, Paul D.P. Pharoah

**Affiliations:** Departments of * Oncology; ‡ Pathology; ¶ Public Health and Primary Care; † Cancer Research UK Cambridge Institute, University of Cambridge; ∥ Department of Pathology, Addenbrooke’s Hospital NHS Foundation Trust, Cambridge; § Medical Research Council Toxicology Unit, Leicester, UK

**Keywords:** breast cancer, tissue microarray, immunohistochemistry, antigenicity

## Abstract

Sectioning a whole tissue microarrray (TMA block) and storing the sections maximizes the number of sections obtained, but may impair the antigenicity of the stored sections. We have investigated the impact of TMA section storage on antigenicity. First, we reexamined existing TMA data to determine whether antigenicity in stored sections changes over time. Component scores for each marker, based on cellular compartment of staining and score-type, were evaluated separately. Residual components scores adjusted for grade, tumor size, and node positivity, were regressed on the number of days storage to evaluate the effect of storage time. Storage time ranged from 2 to 1897 days, and the mean change in antigenicity per year ranged from −0.88 (95% confidence interval, −1.11 to −0.65) to 0.035 (95% confidence interval, 0.016-0.054). Further analysis showed no significant improvement in the fit of survival models if storage time adjusted scores were included in the models rather than unadjusted scores. We then compared 3 ways of processing TMA sections after cutting—immediate staining, staining after 1 year, and staining after 1 year coated in wax—on the immunohistochemistry results for: progesterone receptor, a routinely used, robust antibody, and MKI67, which is generally considered less robust. The progesterone receptor scores for stored sections were similar to those for unstored sections, whereas the MKI67 scores for stored sections were substantially different to those for unstored sections. Wax coating made little difference to the results. Biomarker antigenicity shows a small decline over time that is unlikely to have an important effect on studies of prognostic biomarkers.

## Abstract 2

Supplemental Digital Content is available in the text.

## BACKGROUND

Tissue microarrays (TMAs) constructed using archival, formalin-fixed, paraffin-embedded pathology material, are a standard tool for investigating tumor biomarkers in large-scale clinical epidemiological studies. The reliability of TMAs in such studies has been investigated primarily by focussing on the number of cores needed from each case to produce results equivalent to those from whole tissue sections.^1^–^3^ However, for biomarkers based on immunohistochemistry (IHC) the quality of staining will depend on a wide range of factors. These include preanalytical variables such as the handling and ischemic time of the fresh tumor sample at the time of surgery, the length of fixation and method of tissue processing used, the duration of storage of the paraffin blocks, and the environment in which they are stored, and the methods for TMA construction, the storage conditions for the TMA, the methods for processing TMA sections; and analytical variables within the protocol for the IHC such as antigen retrieval and staining times. The research laboratory often has little control over the initial preanalytical steps, however they can optimize the processes involved in TMA construction, sectioning, and staining. To maximize the number of usable sections available from a single TMA it is common to cut multiple sections at a time to avoid loss of tissue from trimming the block on multiple occasions, and to store the sections for future IHC. However, the efficiency of this approach needs to be balanced against the potential for loss of antigenicity over time due to oxidation of the cut sections.

Fergenbaum et al^4^ showed that antigenicity declined over 6 months for breast TMA sections cut and stored at room temperature. Beckstead^5^ evaluated a specialized fixative solution of zinc salts prepared in a Tris-Ca buffer and found antigen preservation was comparable with frozen sections in blocks stored for up to 3 years, but did not investigate the storage of cut sections. However, the utility of this method is limited because TMAs are usually constructed from archival pathology material so the fixation of the original donor blocks is beyond the control of the research team. Others have reported that coating cut sections in paraffin wax followed by storage in a nitrogen dessicator preserved antigenicity for up to 3 months.^6^ Although this may be a useful approach, specialized equipment for storage of cut sections is not available in many research facilities.

We have generated a large TMA resource that has been used in several large-scale biomarker studies in both breast and ovarian cancer.^7^–^10^ We routinely cut multiple sections from a single TMA block and store these sections for a variable time after cutting. The aim of this study was to evaluate the impact of long-term section storage on the antigenicity of multiple markers. The effect on antigenicity of dipping a freshly cut and mounted section in paraffin wax before long-term storage was also assessed.

## METHODS

### Patient Data

We used data generated from TMAs constructed using archival tumor material from patients in the Study of Epidemiology and Risk factors in Cancer Heredity (SEARCH) breast cancer study, a population-based study of breast cancer. Women diagnosed since 1996 with invasive breast cancer before age 70 in the region served by the National Cancer Registration Service Eastern Office (formerly Eastern Cancer Registration and Information Centre) were eligible to participate. To date, over 13,000 women with breast cancer have enrolled in SEARCH. Information on tumor size, node status, and grade was available from the medical records. In addition, vital status, cause of death, and follow-up time data were available from ECRIC. Archival pathology material from 4125 of these patients has been retrieved from multiple hospital pathology departments across the region for TMA construction. SEARCH has ethical approval from the Multicentre Research Ethics Committee and all the participants have provided informed written consent for their pathology material to be used.

### **TMA Construction, Staining**, **and Scoring**

TMAs were constructed using donor pathology blocks taken from patients under the age of 70 years with invasive breast cancer. Core selection was guided by hematoxylin and eosin–stained slides marked by a pathologist (H.R.A., J.L.Q., or E.P.) for invasive carcinoma. One hundred seventy-two 0.6 mm cores were arrayed in each TMA plus 10 orientation cores; each tumor is represented by a single 0.6 mm core in a TMA constructed from paraffin-embedded tissue blocks. After construction the TMAs were heated to 42°C for 30 minutes and cooled to room temperature 3 times. TMA sections 3 μm thick were cut and mounted on glass slides. These sections were stored for a variable length of time before IHC. Sections were dewaxed in clearene and rehydrated through graded alcohols. IHC was performed using a Bond-Max Autostainer (Leica, UK) as detailed below.

Existing IHC data were available for ALDH1A1, ALDH1A3,^11^ AURKA, GMNN, MKI67, MCM2, PLK1,^7^,^12^ ER, progesterone receptor (PGR), EGFR, CK5/6,^13^ ASMA, CK14,^14^ CDH1,^15^ GATA3,^9^ AR, CTNNB1, FGFR2, FOXP3, KIT, MAP3K1, MYB, NAT1, PDCD4, PTEN, SLC7A5, TP53 (unpublished data) together with date of TMA sectioning and date of IHC processing. Details of reagents and antigen retrieval conditions are summarized in Supplementary Table 1, Supplemental Digital Content, http://links.lww.com/AIMM/A64. The Ariol platform (Genetic Limited, UK) was used to scan the stained slides and the resulting images were used for scoring. The markers were scored for one or more cellular compartment (nucleus, cytoplasm, and membrane) and some markers were scored for both intensity of staining and proportion of cells staining positive. Details of the scoring system for all markers are provided in Supplementary Table 2, Supplemental Digital Content, http://links.lww.com/AIMM/A64. Scoring was carried out by a pathologist (H.R.A., J.L.Q., E.P.) or by a specially trained oncologist (S.-J.D.). In total there were 54 unique combinations of marker, cell compartment, and score-type (component scores).

To evaluate the effect of storage method on antigenicity we used four 3-μm sections cut from each of 2 TMAs and placed onto SuperFrost plus microscope slides. Two of these were dipped in paraffin wax and 2 left exposed to the air. They were then stored in the dark at ambient temperature for 12 months. After 12 months 2 more sections were cut from each TMA and placed onto SuperFrost plus microscope slides, giving a total of 6 sections from each TMA; 2 dipped in paraffin wax and stored for 12 months, 2 exposed to the air and stored for 12 months, and 2 freshly cut (referred to as dipped, undipped, and fresh). The 6 sections were all stained at the same time for PGR and MKI67 using a Leica Bond-Max Autostainer. We used a mouse monoclonal antibody for PGR (DAKO catalog number M3569, lot number 00533, clone, PgR636, isotype IgG1). This was diluted in Bond diluent to a concentration of 1:50. For MKI67 a mouse monoclonal antibody was used (DAKO catalog number M2740, lot number 00027229, clone MIB-1, isotype IgG1). This was diluted using Bond diluent to a concentration of 1:200.

All the cores were scored once by the same pathologist (H.R.A.) for intensity on a 4-point scale (0=no staining, 1=weak, 2=moderate, and 3=strong), and for proportion of positive nuclei on a 6-point scale (0=0%, 1=<1%, 2=1 to <10%, 3=10 to <34%, 4=34 to <67%, 5=67 to <100%).

### Statistical Methods

We evaluated the potential association between IHC scores and storage time using linear regression. However, marker expression is known to vary by tumor characteristics such as tumor size, tumor grade, and node status. This may attenuate any association with storage time. We therefore carried out a linear regression for each component score against tumor grade, tumor size, and number of nodes positive and estimated a residual for each component score for each TMA core (equivalent to the score adjusted for grade, size, and node status). Association between marker expression and number of days between sectioning and staining was then carried out by linear regression of the component score residuals against section storage time. The scores for dipped, undipped, and fresh cores were compared using a kappa statistic.

To evaluate the importance of any loss of antigenicity over time we evaluated the prognostic significance of each component score in 2 multivariable Cox proportional hazards models of breast cancer–specific survival. In 1 model the observed score was used and in the second model the score adjusted for storage time was used. Grade, size, and node status were included as covariates in both models. The models were compared using the model log likelihood statistics.

## RESULTS

**FIGURE 1:**
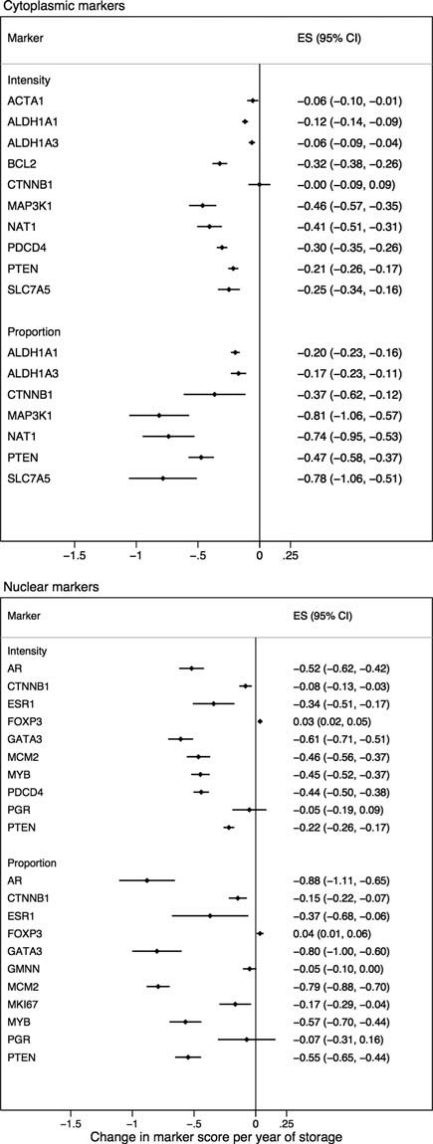
Change in component marker scores per year of storage time.

The number of cases with IHC and clinical data ranged from 306 for CASP8 to 2467 for TP53. Scatterplots of the score residuals against storage time are shown in Supplementary Figure 1, Supplemental Digital Content, http://links.lww.com/AIMM/A64. The mean change in antigenicity over time for each marker component is shown in Table 1. All but 2 of the marker components showed a decline in antigenicity over time and for 40 of them the decline was significant at a nominal *P*<0.05. However, the loss of antigenicity was small. The biggest effect was seen for the proportion score for nuclear androgen receptor, which declines on an average by 0.88 units per year. Figure 1 shows the mean change in antigenicity for cytoplasmic and nuclear markers stratified by intensity and proportion scores. The decline in antigenicity for cytoplasmic proportion, nuclear intensity, and nuclear proportion scores was similar, but the decline in cytoplasmic intensity scores was greater. The results of the Cox regression models carried out on each marker component indicated that there was no improvement in the fit of the model when storage time was included (data not shown).

**TABLE 1:**
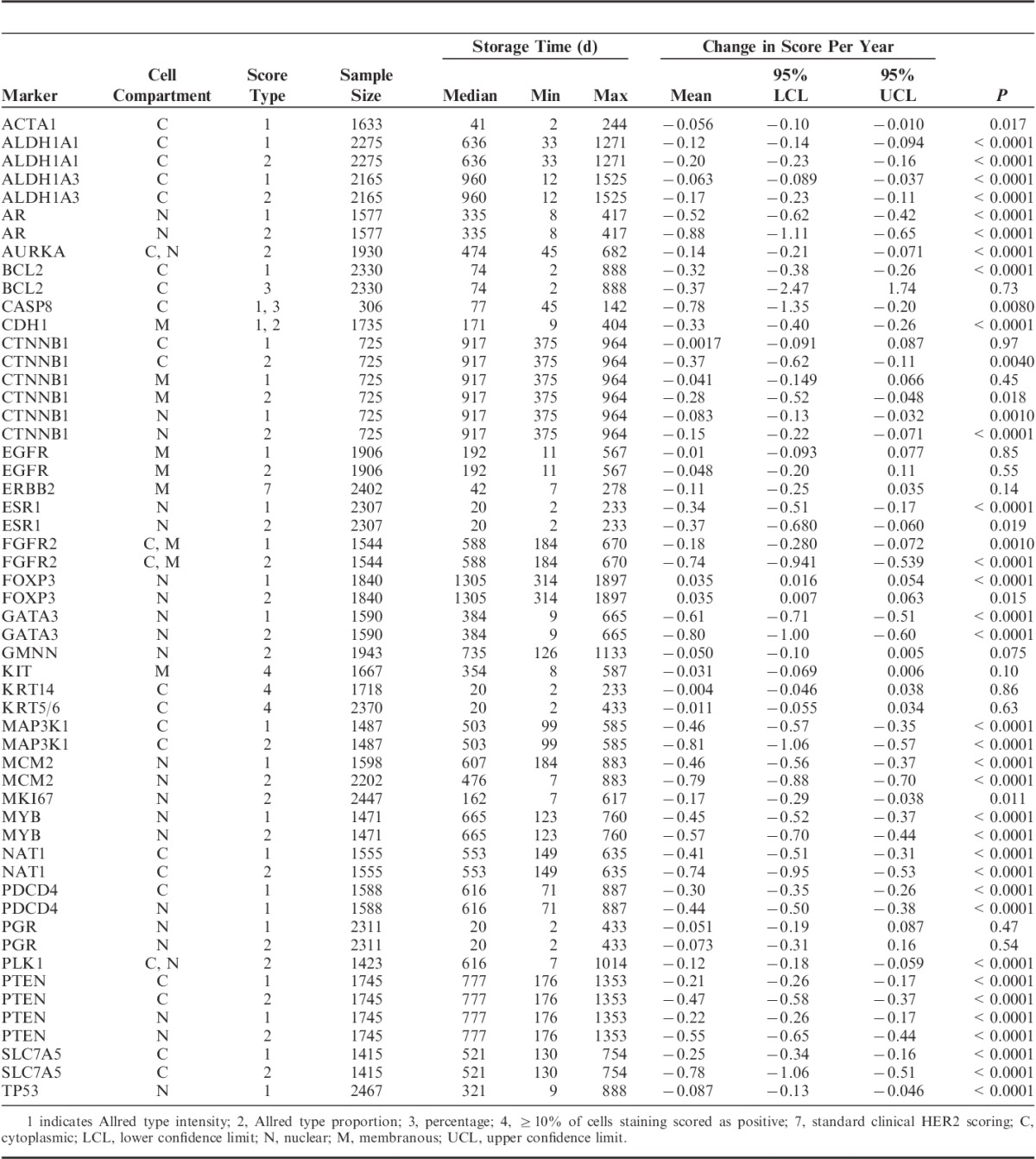
Change in Antigenicity Over Time for Each Marker Component

**FIGURE 2:**
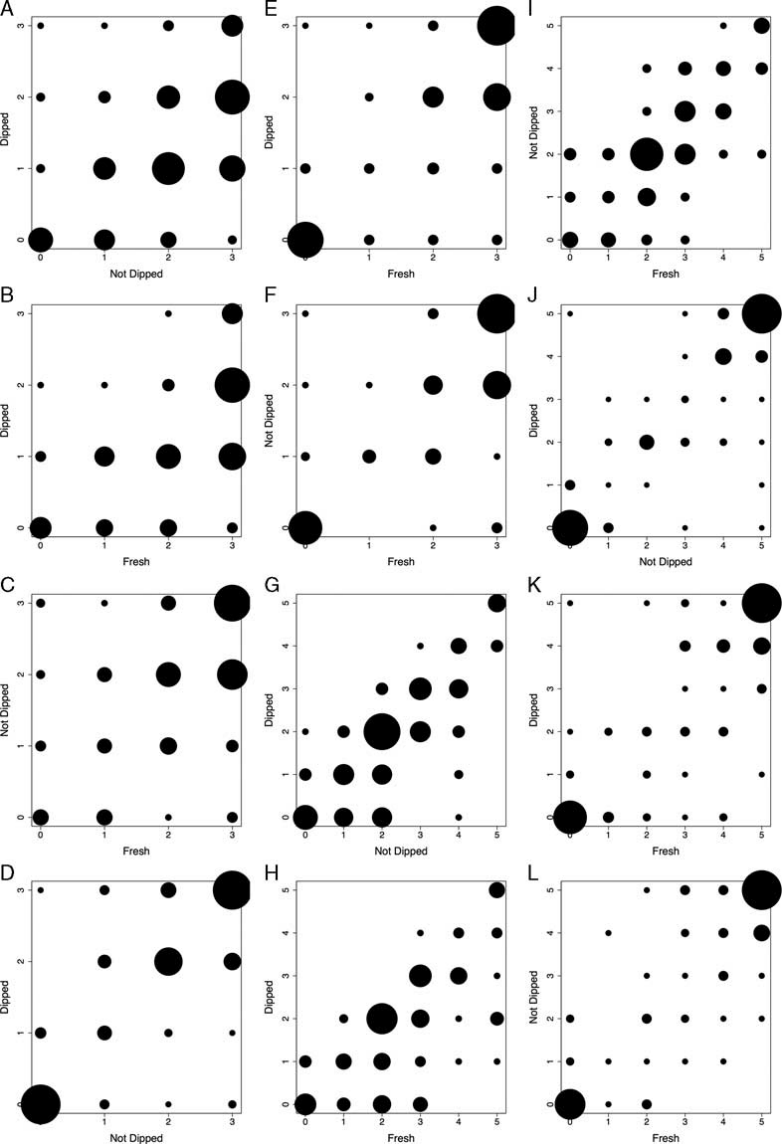
Weighted scatterplot of component marker scores by processing method. A, MKI67 intensity dipped versus not-dipped. B, MKI67 intensity dipped versus fresh. C, MKI67 intensity not-dipped versus fresh. D, PGR intensity dipped versus not-dipped. E, PGR intensity dipped versus fresh. F, PGR intensity not-dipped versus fresh. G, MKI67 proportion dipped versus not-dipped. H, MKI67 proportion dipped versus fresh. I, MKI67 proportion not-dipped versus fresh. J, PGR proportion dipped versus not-dipped. K, PGR proportion dipped versus fresh. L, PGR proportion not-dipped versus fresh. PGR indicates progesterone receptor.

We compared the IHC scores for PGR and MKI67 for dipped, undipped, and fresh sections. Typical examples of the IHC of the same core processed by these methods are shown in Supplementary Figure 2, Supplemental Digital Content, http://links.lww.com/AIMM/A64. Weighted scatterplots for each comparison pair of scores of these results are shown in Figure 2 (n=133 to 153 paired scores). For fresh compared with dipped sections, weighted kappa values of 0.89, 0.80, 0.64, and 0.44 were obtained for PGR proportion, PGR intensity, MKI67 proportion, and MKI67 intensity, respectively. The equivalent values were 0.91, 0.80, 0.72, and 0.54 for fresh compared with undipped sections and for dipped compared with undipped the scores were 0.93, 0.86, 0.77, and 0.45. If the commonly used scale of: 0.01 to 0.20=slight agreement, 0.21 to 0.40 fair agreement, 0.41 to 0.60 moderate agreement, 0.61 to 0.80 substantial agreement, 0.81 to 0.99 almost perfect agreement, is applied all the kappa values obtained indicated at least moderate agreement.

## DISCUSSION

The results obtained by these analyses show that there is a significant but small decline in antigenicity with increasing storage time; however, when scores adjusted for storage time are used in multivariable survival time Cox regression models there is no improvement in the fit of the model compared with a similar model with unadjusted scores. This may be because antigenicity simply declines proportionately across all IHC subtypes.

The results of the experiment using 2 different storage methods of TMA sections after cutting and freshly cut sections, provide evidence that when using a robust IHC marker such as PGR, scores obtained from sections cut and stored for a year were similar to those obtained from freshly cut sections. However, for MKI67, the less robust marker, the correlation between scores for freshly cut and stored sections was weaker. The method of storage made little difference to this. The difference in the effect of storage on PGR and MIKI67 was also found in the analysis of the effect of storage time on the SEARCH TMAs with a reduction in the proportion score for PGR of 0.073 per year compared with a reduction of 0.17 for the MKI67 proportion score (Table 1).

There are many possible methods that might be used to reduce the time dependent decline in antigenicity, but the utility of evaluating these is likely to be limited given the small decline in antigenicity for sections stored for up to a year. A 1 year storage time is longer than that evaluated in previous research. Nevertheless, many pathology and/or research facilities store cut sections for much longer. Given that the observations in this study may not be linear with increasing lengths of time testing of the impact of storage time is an important parameter that investigators involved in biomarker studies should address in their study design.

Our data suggest that medium-term storage time for sections cut from TMAs is not a major factor in the reliability of most IHC biomarkers. In large-scale studies evaluating multiple biomarkers, the cutting of multiple sections from a TMA for future staining is an efficient use of a scarce resource that will not affect adversely the findings of biomarker studies.

## Supplementary Material

SUPPLEMENTARY MATERIAL
