# Supplementary material for: Decline in Antigenicity of Tumor Markers by Storage Time Using Pathology Sections Cut From Tissue Microarrays
Source: Appl Immunohistochem Mol Morphol. 2016 Mar 10;24(3):221–6. doi: 10.1097/PAI.0000000000000172 (PMC4892716; doi:10.1097/PAI.0000000000000172)
Supplement: SUPPLEMENTARY MATERIAL [file pai-24-221-s001.pdf]

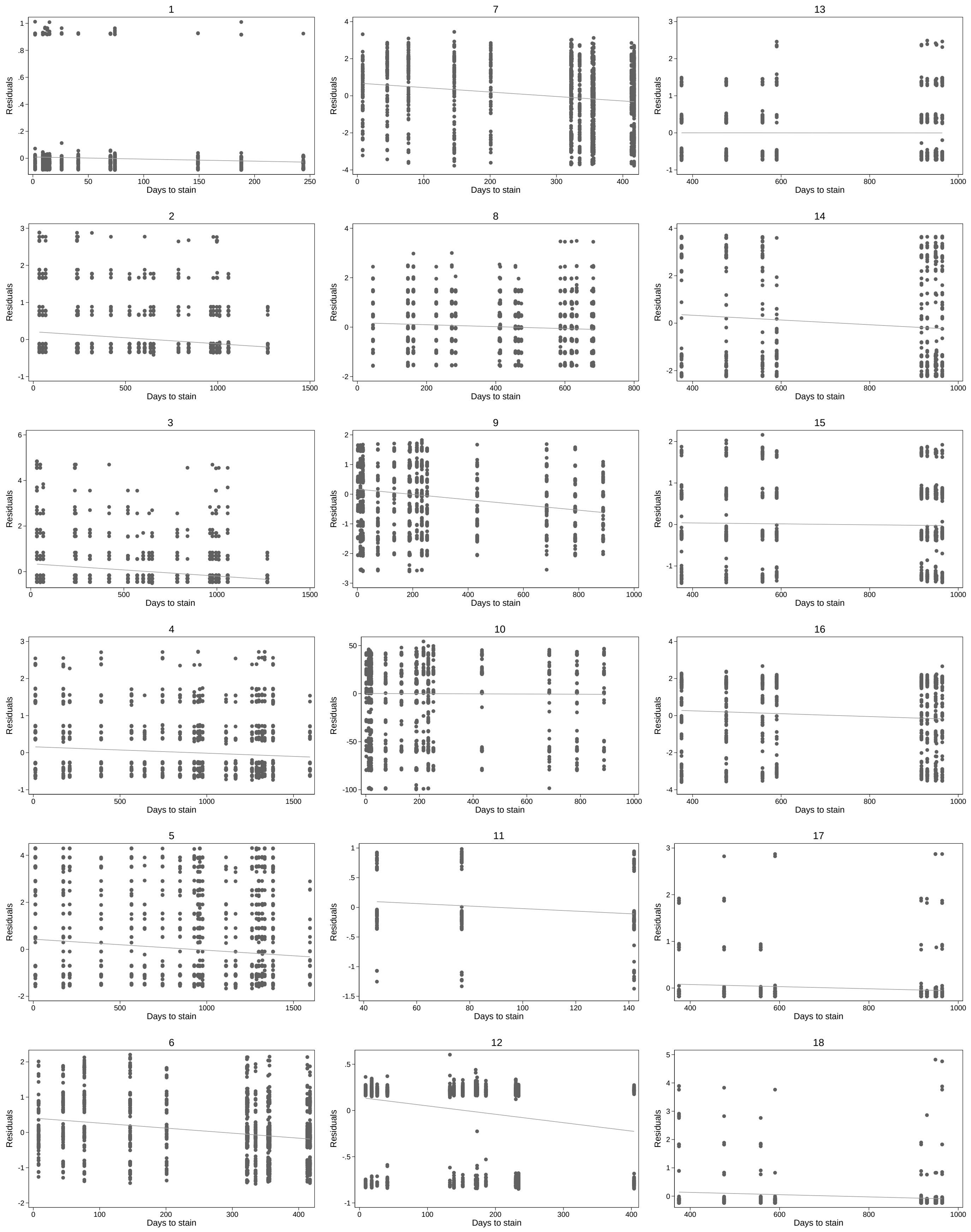

1; ACTA1,cytoplasmic,1 2; ALDH1A1,cytoplasmic,1 3; ALDH1A1,cytoplasmic,2
4; ALDH1A3,cytoplasmic,1 5; ALDH1A3,cytoplasmic,2 6; AR,nuclear,1
7; AR,nuclear,2 8; AURKA,cytoplasmic/nuclear,2 9; BCL2,cytoplasmic,1
10; BCL2,cytoplasmic,3 11; CASP8,cytoplasmic,13 12; CDH1,membranous,12
13; CTNNB1,cytoplasmic,1 14; CTNNB1,cytoplasmic,2 15; CTNNB1,membranous,1
16; CTNNB1,membranous,2 17; CTNNB1,nuclear,1 18; CTNNB1,nuclear,2

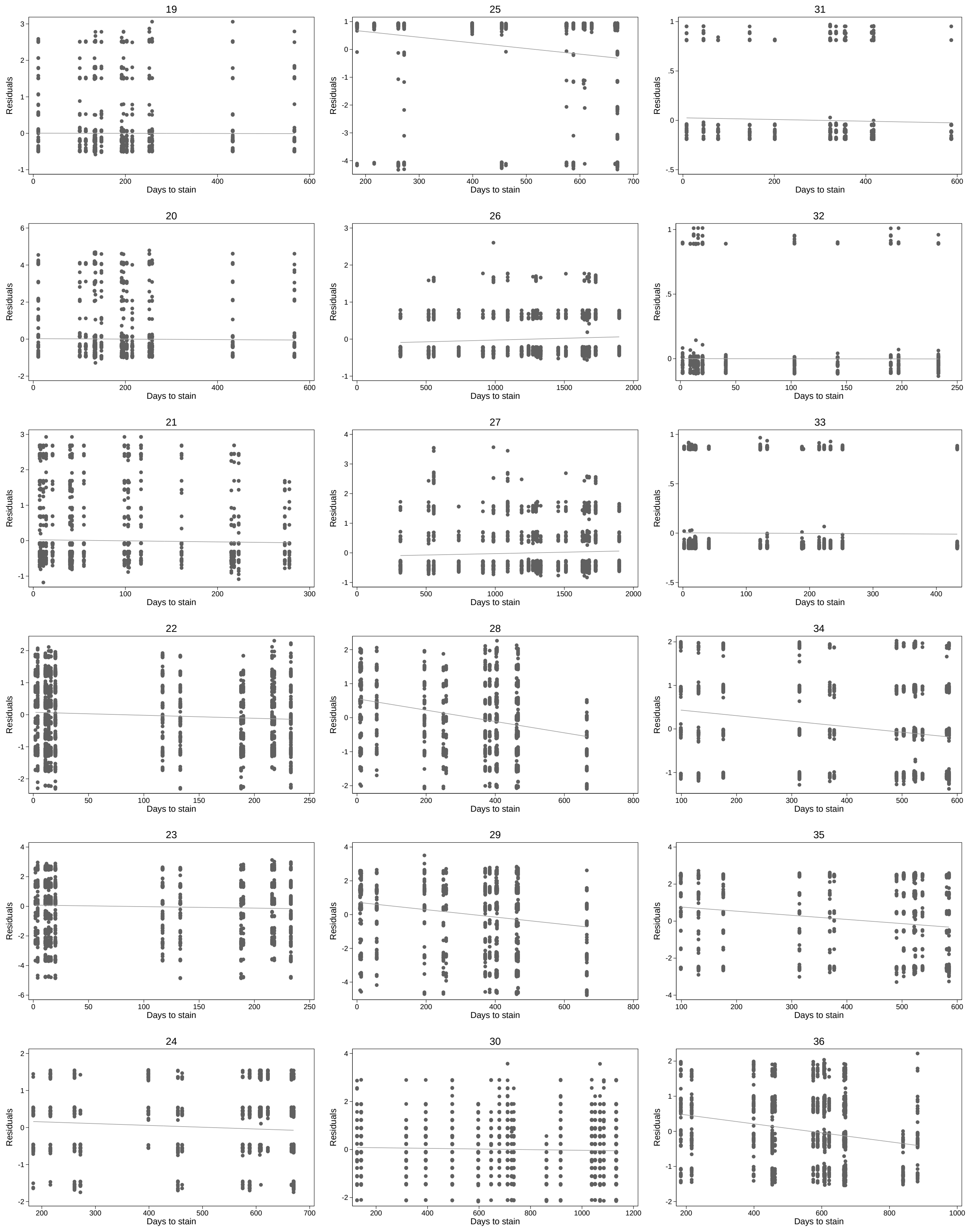

19; EGFR,membranous,1 20; EGFR,membranous,2 21; ERBB2,membranous,7
22; ESR1,nuclear,1 23; ESR1,nuclear,2 24; FGFR2,cytoplasmic/membranous,1
25; FGFR2,cytoplasmic/membranous,2 26; FOXP3,nuclear,1 27; FOXP3,nuclear,2
28; GATA3,nuclear,1 29; GATA3,nuclear,2 30; GMNN,nuclear,2 31; KIT,membranous,4
32; KRT14,cytoplasmic,4 33; KRT5/KRT6,cytoplasmic,4
34; MAP3K1,cytoplasmic,1 35; MAP3K1,cytoplasmic,2 36; MCM2,nuclear,1

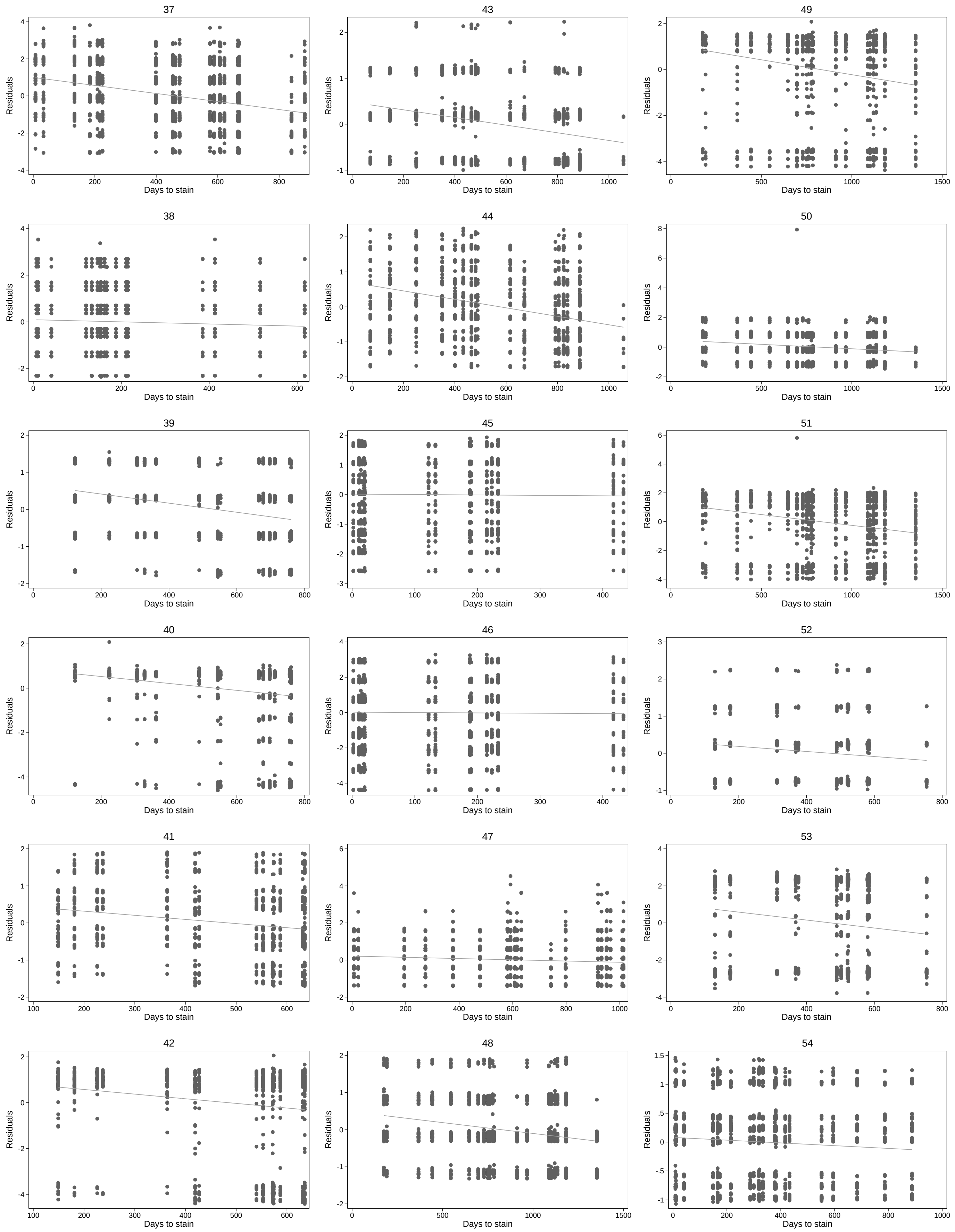

37; MCM2,nuclear,2 38; MKI67,nuclear,2 39; MYB,nuclear,1 40; MYB,nuclear,2
41; NAT1,cytoplasmic,1 42; NAT1,cytoplasmic,2 43; PDCD4,cytoplasmic,1
44; PDCD4,nuclear,1 45; PGR,nuclear,1 46; PGR,nuclear,2 47; PLK1,cytoplasmic/nuclear,2
48; PTEN,cytoplasmic,1 49; PTEN,cytoplasmic,2 50; PTEN,nuclear,1
51; PTEN,nuclear,2 52; SLC7A5,cytoplasmic,1 53; SLC7A5,cytoplasmic,2 54; TP53,nuclear,11

Supplementary Figure 2: Six examples of the same core with two sections stained for PR. One section stained immediately after sectioning and the other stained after storing for one year

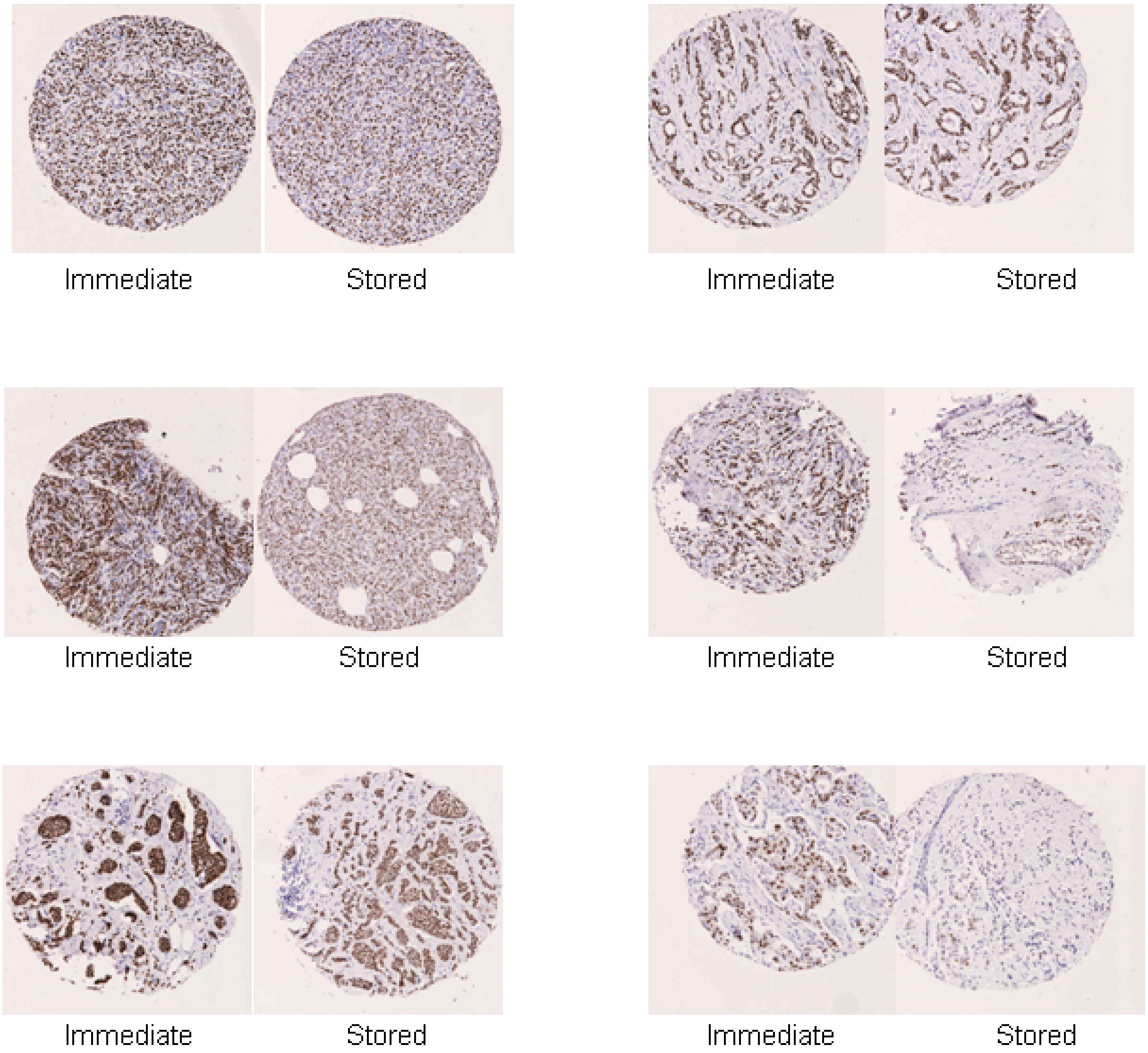

Official Symbol	Manufacturer	Antibody label	Target Species	Raised Species	Clonality	Clone	Conc	Diluent
ACTA1	Sigma	Primary	Human	Mouse	Monoclonal	1A4	1:2000	Bond
ALDH1A1	Atlas	Primary	Humna	Rabbit	Polyclonal		01:50	Citrate Buffer pH6
ALDH1A3	Abgent	Primary	Human	Rabbit	Polyclonal		1:25	Sanger 300mM
AR	Novocastra	Primary	Human	Mouse	Monoclonal	AR27	1:50	Bond
AURKA	Novocastra	Primary	Human	Mouse	Monoclonal	JLM28	1:25	Sanger
BCL2	DAKO	Primary	Human	Mouse	Monoclonal	124	1:200	Bond
CASP8	Novocastra	Primary	Human	Mouse	Monoclonal	11B6	1:50	Bond
CDH1	DAKO	Primary	Human	Mouse	Monoclonal	NCH-38	1:25	Bond
CTNNB1	BD Biosciences	Primary	Human	mouse	Monoclonal	31/07/2014	1:5000	
EGFR	Zymed	Primary	Human	Mouse	Monoclonal	31 G7	1:25	Bond
ESR1	Novocastra	Primary	Human	Mouse	Monoclonal	6F11/2	1:70	Bond
FGFR2	Sigma	Primary	Human	Rabbit	Polyclonal		1:50	Bond
FOXP3	Abcam	Primary	Human	Mouse	Monoclonal	236A/E7	1:100	Bond
GATA3	Santa Cruz	Primary	Human	Mouse	Monoclonal	(HG3-31)	1:80	Bond
GMNN	Novocastra	Primary	Human	Mouse	Monoclonal	EM6	1:50	Bond
KIT	DAKO	Primary	Human	Rabbit	Polyclonal		1:200	Bond
KRT14	Novocastra	Primary	Human	Mouse	Monoclonal	LL002	1:20	Bond
KRT5/KRT6	DAKO	Primary	Human	Mouse	Monoclonal	D5/16 B4	1:50	Bond
MAP3K1	Santa Cruz	Primary	Human	Mouse	Monoclonal	F-11	1:1000	Bond
MCM2	Novocastra	Primary	Human	Mouse	Monoclonal	CRCT2.1	1:50	Bond
MKI67	DAKO	Primary	Human	Mouse	Monoclonal	MIB-1	1:200	Bond
MYB	Upstate	Primary	Human	Mouse	Monoclonal	1-1	1:250	Bond
NAT1	BD Biosciences	Primary	Human	Mouse	Monoclonal	35	1:100	Sanger
PDCD4	Abcam	Primary	Human	Rabbit	Monoclonal	EPR3431	1:1500	Bond
PGR	DAKO	Primary	Human	Mouse	Monoclonal	PgR 636	1:50	Bond
PLK1	Upstate	Primary	Human	Mouse	Monoclonal	35-206	1:7500	Sanger
PTEN	DAKO	Primary	Human	Mouse	Monoclonal	6H2.1	1:200	Bond
SLC7A5	Novus Biologicals	Primary	Human	Rabbit	Polyclonal		1:100	Bond
TP53	DAKO	Primary	Human	Mouse	Monoclonal	DO-7	1:1000	Bond

Variable Code	Score Type	Description
1	Intensity	Allred type. 0 = none, 1 = weak, 2 = moderate, 3 = strong
2	Proportion	Allred type 0 = 0%, 1 = <1%, 2 = 1 - <10%, 3 = 10 - <34%, 4 = 34 - <67%, 5=67-<100%
3	PercentagePositive	Direct recrd of percentage of cells staining.
4	10%CutOff	≥10%of cells staining = positive
7	HER2	0 = none or <10%, 1 = weak and incomplete in>10%, 2 = moderate complete >10%, 3 = strong >10%
